# Supplementary material for: Mitochondrial stress response triggered by defects in protein synthesis quality control
Source: Life Sci Alliance. 2019 Jan 25;2(1):e201800219. doi: 10.26508/lsa.201800219 (PMC6348486; doi:10.26508/lsa.201800219)
Supplement: Supplementary file 1 [file LSA-2018-00219_Table_S1.pdf]

Amplicon	Detected Variant SNP & InDel	Allele frequency on rCRS	Read depth at SNP	9205.WT Frequency on 9205del.TA reference*
1	8251.G>A	86.28%	182141	
	8472.C>T	76.25%	203782	
	8836.A>G	93.36%	233309	
	8860.A>G	95.18%	228059	
	9205.delTA	91.14%	232620	2.94%
	9335.C>T	91.25%	233553	
	10238.T>C	94.55%	219929	
2	7028.C>T	92.04%	183004	
	8251.G>A	86.64%	169134	
	8472.C>T	74.35%	162972	
	8836.A>G	93.54%	174559	
	8860.A>G	95.06%	170110	
	9205.delTA	91.26%	154477	2.96%
	9335.C>T	89.78%	166329	
3	9205.delTA	94.08%	294628	1.93%
	9335.C>T	92.65%	311329	
	10238.T>C	93.83%	290998	
